# A Practical Treatise on the Efficacy of Stizolobium, or Cowhage, Internally Administered in Diseases Occasioned by Worms. To Which Are Added Observations on Other Anthelmintic Medicines of the West Indies

**Published:** 1785

**Authors:** 


III. *A practical Treatise on the Efficacy of Stizolobium, or Cowhage, internally administered in Diseases occasioned by Worms. To which are added Observations on other anthelmintic Medicines of the West Indies. By* William Chamberlaine, *Surgeon.* 8vo. Murray, *London,* 1784. 77 pages.

THIS little work merits attention, as it appears to be written with candour, and tends to confirm the efficacy of a medicine which, though recommended by different writers as a powerful anthelmintic in cafes of *lumbrici*, has hitherto, we believe, been but little employed in this country.

Stizolobium*, or Cowhage, as it is commonly called by Europeans, is the *Dolichos pruriens* of Linnæus. Of this plant, which is a native both of the Eaft and Weft Indies, an accurate defcription is given by Mr. Kerr in the fecond volume of the Medical Commentaries.

The part ufed is the fetaceous hairy fubftance, growing on the outfide of the pod, which is directed to be fcraped off, and mixed with ho-

* So named by Dr. Browne in his Natural Hiftory of Jamaica.

ney, melaſſes, or ſyrup, to the conſiſtence of a thin electuary. Adminiſtered in this form, the medicine ſeems to have merely a mechanical operation on the worms, as a tincture or decoction of the ſpiculæ are found to be of no uſe.

Of this electuary, a tea-ſpoonful, given in a morning, faſting, is ſaid to be a ſufficient doſe for young children; but to adults our author gives one or even two table-ſpoonfuls. This may be repeated two or three mornings; but in general, he obſerves, there is ſeldom occaſion to go beyond the third doſe, and a gentle purge commonly completes the cure.

In praiſe of this medicine Mr. Chamberlaine does not go ſo far as to ſay that he has never known it to fail; but he aſſerts that he has experienced more certain good effects, and fewer ill conſequences from it, than from any other medicine he has employed with the ſame intention.

Seven caſes, ſelected from a much greater number, in which the cowhage is ſaid to have been adminiſtered with equal ſucceſs, are given as proofs of its ſafety and efficacy; as is likewiſe a letter to the author from Mr. Neil Stewart, ſurgeon in Jamaica, who confirms his opinion opinion of this medicine, and thinks "it may "be looked on as a more certain ſpecific in "worm complaints than the Peruvian bark is "in the cure of intermittents."

Mr. Bancroft, who ſpeaks of this remedy in his Natural Hiſtory of Guiana, confines its uſe to caſes of the round worm; as does likewiſe Mr. Chamberlaine in the pamphlet now before us; but ſince its publication (as he has had the goodneſs to inform us) he has convinced himſelf by repeated trials that it is equally efficacious in caſes of aſcarides.

